# Supplementary figures and images for: The diagnostic tests and functional outcomes of acute ischemic stroke or transient ischemic attack in young adults: A 4-year hospital-based observational study
Source: PLoS One. 2023 Oct 4;18(10):e0292274. doi: 10.1371/journal.pone.0292274 (PMC10550126; doi:10.1371/journal.pone.0292274)

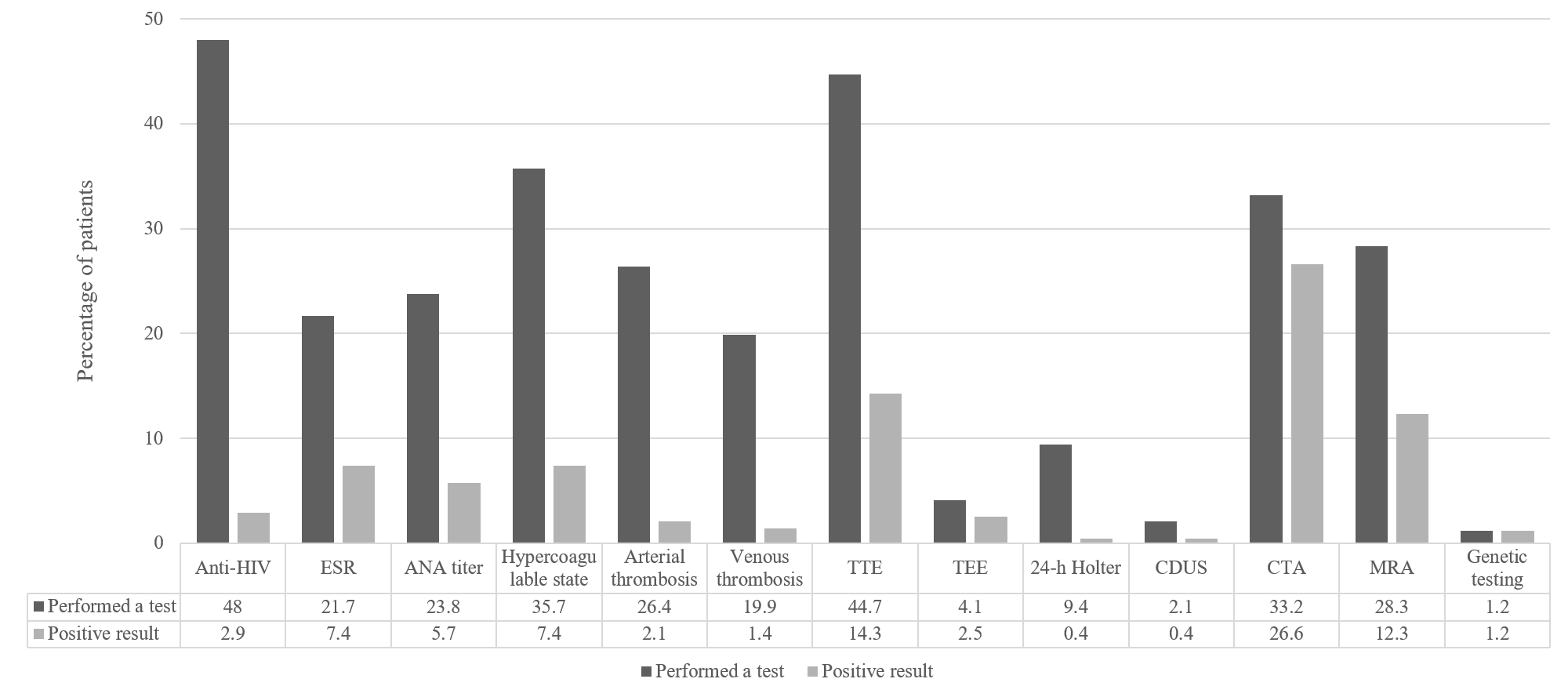

Supplement: S1 Fig — ANA, antinuclear antibody; CDUS, carotid doppler ultrasonography; CTA, computed tomography angiography; ESR, erythrocyte sedimentation rate; HIV, human immunodeficiency virus; MRA, magnetic resonance angiography; TEE, transesophageal echocardiography; TTE, transthoracic echocardiography. (TIF) [file pone.0292274.s001.tif]
