# Supplementary material for: The diagnostic tests and functional outcomes of acute ischemic stroke or transient ischemic attack in young adults: A 4-year hospital-based observational study
Source: PLoS One. 2023 Oct 4;18(10):e0292274. doi: 10.1371/journal.pone.0292274 (PMC10550126; doi:10.1371/journal.pone.0292274)
Supplement: Attachment — Submitted filename: Response to Reviewers.docx [file pone.0292274.s002.docx]

**Manuscript number: PONE-D-23-05766**

**Title: The diagnostic tests and functional outcomes of acute ischemic stroke or transient ischemic attack in young adults: A 4-year hospital-based observational study**

**Responses to Reviewers**

We would like to thank the editor and the reviewers for all the valuable comments and suggestions. We have revised the manuscript based on these constructive suggestions. We believe the revised manuscript is now greatly improved after the revisions have been made, as suggested by the reviewers and the editor.

**Responses to Editorial Corrections**

**Q1:** Please ensure that your manuscript meets PLOS ONE's style requirements, including those for file naming.

**A1:** We revised the manuscript and file naming following PLOS ONE’s style requirement.

**Q2:** We note that you have indicated that data from this study are available upon request. PLOS only allows data to be available upon request if there are legal or ethical restrictions on sharing data publicly.

**A2:** We would like to thank the editor for the correction. We revised Data Availability Statement based on the information on unacceptable data access restrictions. **Now it read** “Data cannot be shared publicly because of ethical issues. Data are available from the Ethics Committee of the Faculty of Medicine, Chiang Mai University (contact via Researchmed@cmu.ac.th) for researchers who meet the criteria for access to confidential data.” (In revised cover letter)

**Responses to Reviewer 1:**

**Q1:** Genetic testing was performed in only 3 pts. A general conclusion about its value could not be concluded and should not be so emphasized**.**

**A1:** We would like to thank the reviewer for the suggestion. We have revised the general conclusion regarding the value of genetic testing in young stroke patients, as suggested by the reviewer. **Now it read** “**Conclusions**: Stroke of other determined etiology remained the common cause of stroke in young adults, and most affected individuals had excellent clinical outcomes. Blood tests for arterial hypercoagulability and noninvasive vascular and cardiac evaluations are encouraged in selected patients to determine the stroke etiology and guide for appropriate preventive strategies.” (Pages 2-3, lines 38-41)

**Q2:**  Complete diagnostic data were only available in 36%. This is a thin database to state conclusions.

**A2:** We would like to thank the reviewer for the valuable suggestion. We rearranged the conclusion of the revised manuscript to be more concise and precise according to a reviewer’s suggestion. **Now it read** “In conclusion, our study has shown Anti-HIV, TTE, hypercoagulable state, particularly APS testing, and noninvasive vascular investigation with CTA are typically conducted in addition to traditional assessments or basic laboratory testing for identifying stroke in young adults. Using these diagnostic tests incorporated with initial investigations following standard or routine blood tests to determine stroke etiologies in properly selected young adult stroke or TIA patients can lead to a clinical benefit, as early diagnosis and treatment can significantly improve patient outcomes.” (Pages 23-24, lines 311-316)

**Q3:** In Conclusion: Stroke could not be diagnosed with TEE. Only causes of stroke could be evaluated.

**A3:** We would like to thank the reviewer for the valuable suggestion. We rearranged the conclusion of the revised manuscript to be more concise and precise according to a reviewer’s suggestion. **Now it read** “**Conclusions**: Stroke of other determined etiology remained the common cause of stroke in young adults, and most affected individuals had excellent clinical outcomes. Blood tests for arterial hypercoagulability and noninvasive vascular and cardiac evaluations are encouraged in selected patients to determine the stroke etiology and guide for appropriate preventive strategies.” (Pages 2-3, lines 38-41) and “In conclusion, our study has shown Anti-HIV, TTE, hypercoagulable state, particularly APS testing, and noninvasive vascular investigation with CTA are typically conducted in addition to traditional assessments or basic laboratory testing for identifying stroke in young adults. Using these diagnostic tests incorporated with initial investigations following standard or routine blood tests to determine stroke etiologies in properly selected young adult stroke or TIA patients can lead to a clinical benefit, as early diagnosis and treatment can significantly improve patient outcomes.” (Pages 23-24, lines 311-316)

**Q4:** Table 1: Is a cause why congestive heart failure in the younger group is more often than in the older?

**A4:** We would like to thank the reviewer for the suggestion. The causes of congestive heart failure in the younger group are currently mentioned in the Results part. **Now it read** “Interestingly, congestive heart failure (CHF) was commonly diagnosed in the younger patient group, in which non-ischemic dilated cardiomyopathy was a certain cause in this population (10.8% vs. 1.3%, *P* = 0.001). Table 1 demonstrates the baseline characteristics and risk factors of the participants, classified into two cohorts.” (Page 8, lines 133-136)

**A5:** Table 2: In the line with Ischemic stroke classification: to which belongs the p-value 0.001?

**Q5:** We would like to thank the reviewer for the helpful suggestion. The P-value 0.001 belongs to the difference among all ischaemic stroke classifications. We performed an additional statistical analysis to test the difference among ischaemic stroke subtypes. **Now it read** “Compared to a younger age group, AIS aged 41-50 had more prevalence of large-artery atherosclerosis (LAA) and small-vessel occlusion (SVO) stroke subtype according to TOAST classification, 21.9% vs. 9.7%, *P* = 0.014 and 27.8% vs. 11.8%, *P* = 0.003, respectively (Fig 2).” (Page 10, lines 145-148) and Table 2 Pages 11-12.

**Q6:** No p-values are given for: large artery arterosclerosis, cardioembolism, small vessel occlusion, stroke from other determined etiology and stroke of indetermined etiology.

**A6:** We apologise for this mistake. We performed statistical analysis to test the difference among ischaemic stroke subtypes, as the reviewer suggested. **Now it read** “Focus on younger patients, AIS patients in the 18-40 age group had a significantly higher proportion of stroke of other determined etiology, 39.8% vs. 25.2%, *P* = 0.016, compared to an older group (Table 2 and Fig 2). The most common cause of ischemic stroke of other determined etiology is a hypercoagulable state (8.2%), and hereditary thrombophilia is commonly diagnosed (4.1%). These findings are associated with a higher proportion of anticoagulant prescriptions in AIS patients in the younger age group (23.7% vs. 13.4%, *P* = 0.04).” (Pages 13-14, lines 168-173) and Table 2 Pages 11-12.

**Q7:** There are patients, who didn´t get a TTE or TEE. Which type of diagnostic workup did they get? Only in 10 patients a TEE was performed. Why? This is a small database to conclude anything about the value of TEE and especially in comparison with TTE.

**A7:** We would like to thank the reviewer for the valuable suggestion. The information regarding TEE and additional discussion on its diagnostic value have been illustrated in the Results and Discussion part of the revised manuscript. **Now it read** “Among cardiac evaluation, transthoracic echocardiography (TTE) was commonly performed in 44.7% (109 of 244) of patients and provided positive results in 32.1% with no difference between gender or age groups. Transesophageal echocardiography (TEE) illustrated diagnostic results in 60% of included patients. Still, its diagnostic yield cannot be generalized clinical implication because it was performed on only ten patients and selected populations with highly suspected positive cardiac causes.” (Page 16, lines 201-206) and “Although TEE has 60% positive test results for patients who were evaluated cardiac sources of stroke or emboli, it was only carried out in 4% of patients in our cohort and might not be an inference to its higher diagnostic yields when compared with other cardiac assessments. This could be explained by the fact that TTE is typically followed by TEE to offer a comprehensive evaluation of abnormal cardiogenic morphology.[15, 16] These noninvasive diagnostic procedures, however, are probably recommended for young individuals due to increasing the detection of vascular dissection and cardiac sources of stroke, especially PFO with or without ASA, which are the common causes of stroke among young adult patients.” (Page 21, lines 257-264)

**Responses to Reviewer 2:**

**Q1:** However, one main aspect of the publication (“the yield of diagnostic tests”) is insufficient, because these test have not been systematically performed (the Authors adequately mention the main limitation of their study: “A standardized stroke workup was not done”). The aspect “yield of diagnostic tests” should be completely omitted.

**A1:** We would like to thank the reviewer for the valuable suggestion. We decided to adjust the study title for suitability and appropriateness with the details of the revised manuscript. **Now it read** “The diagnostic tests and functional outcomes of acute ischemic stroke or transient ischemic attack in young adults: A 4-year hospital-based observational study” (Page 1, lines 1-2) In addition, the Abstract and Discussion parts have been rearranged by adding additional scientific information to enhance the quality of the work. **Now it read** “**Background and objectives**: Ischemic strokes in young adults have been a significant concern due to various potential etiologies and had substantial clinical and public health impacts. We aimed to study the diagnostic tests, etiologies, and functional outcomes of acute ischemic stroke (AIS) and transient ischemic attack (TIA) in young adult patients.

**Methods**: The data were retrieved from the Chiang Mai University Hospital Stroke Registry between January 2018 and December 2021. Consecutive AIS or TIA patients were included if they were 18-50 years and had no stroke mimics. Study outcomes were proportions of positive diagnostic tests, and 90-day modified Rankin Scale (mRS).

**Results**: Of 244 enrolled patients, 59.0% (n = 144) were male, and 38.1% (n = 93) were aged 18-40, classified as the younger age group. There was a high incidence of diabetes (24.5%) and dyslipidemia (54.3%) among patients aged 41-50, associated with small-vessel occlusion and large-artery atherosclerosis stroke classification in this age group. Patients aged 18-40 years had more other determined etiologies (39.8%), with hypercoagulability (8.2%), arterial dissection (7.8%), and cardiac sources (6.6%) being the first three causes, which were associated with higher anticoagulant treatment. The cerebrovascular study, cardiac evaluation using echocardiography, and antiphospholipid syndrome testing were commonly performed, of which computed tomography angiography provided a high proportion of positive results (80.3%). 76.3% of young adult patients had excellent functional outcomes (mRS 0-1) with a median mRS of 0 (interquartile range 0-1) at 90-day follow-up.

**Conclusions**: Stroke of other determined etiology remained the common cause of stroke in young adults, and most affected individuals had excellent clinical outcomes. Blood tests for arterial hypercoagulability and noninvasive vascular and cardiac evaluations are encouraged in selected patients to determine the stroke etiology and guide for appropriate preventive strategies.” (Pages 2-3, lines 19-41), “On the contrary, the diagnostic yields of these investigations, especially compared to others, should be interpreted cautiously because it was not entirely performed in all cohorts.” (Page 20, lines 230-232), and “The specialized investigation, genetic testing for CADASIL (NOTCH3 mutation) and MELAS is often conducted in young adult patients with clinical clues compatible with these stroke syndromes. Additionally, these diagnostic procedures are usually done as a step-by-step approach in patients who have undergone all other investigations yet have not found any etiology, which only included three patients in our study. As a result, the value of these diagnostic yields typically varied between the studies. Dong Y.’s study conducted in two hundred eighteen consecutive patients found the overall frequency of CADASIL mutations in lacunar stroke to be less than 0.5%.[11] Therefore, the difference in the suspected population might affect the inconsistent results of the diagnostic yield of genetic tests in young individuals who suffer from acute ischemic stroke or TIA.

The vascular study of cerebral vessels and neck, including CTA, magnetic resonance angiography (MRA), or carotid doppler ultrasonography (CDUS), provides details of cerebral vasculatures and guides therapeutic interventions in acute stroke settings and long-term secondary stroke prevention.[12, 13] Therefore, noncontrast computed tomography (NCCT) and CTA brain have been utilized as standard stroke neuroimaging in acute stroke patients.[14] Among these investigations, our finding reveals that CTA represents a higher proportion of positive results compared to MRA and CDUS. Limitations and accessibility of MRA and operator-dependent of CDUS might be the answer for our cohort that these vascular imagings are lower number performed and proportion of positive tests. Similar to Ji R. *et al.*, which also found a superiority of vascular imaging, CTA, in young individuals.[15]” (Pages 20-21, lines 234-253)

**Q2:** The final conclusion in the abstract “Our study show a high-yield diagnostic test of CTA and TEE for deteremining stroke etiology in young adults. Prompt diagnosis and treatment of stroke can lead to better patient outcomes, making using these tests a valuable strategy” is completely unproven and should be omitted.

**A2:** We would like to thank the reviewer for the helpful suggestion. As the reviewer suggested, the conclusion of the revised manuscript has been rewritten to be more concise and precise. **Now it read** “**Conclusions**: Stroke of other determined etiology remained the common cause of stroke in young adults, and most affected individuals had excellent clinical outcomes. Blood tests for arterial hypercoagulability and noninvasive vascular and cardiac evaluations are encouraged in selected patients to determine the stroke etiology and guide for appropriate preventive strategies.” (Pages 2-3, lines 38-41) and “In conclusion, our study has shown Anti-HIV, TTE, hypercoagulable state, particularly APS testing, and noninvasive vascular investigation with CTA are typically conducted in addition to traditional assessments or basic laboratory testing for identifying stroke in young adults. Using these diagnostic tests incorporated with initial investigations following standard or routine blood tests to determine stroke etiologies in properly selected young adult stroke or TIA patients can lead to a clinical benefit, as early diagnosis and treatment can significantly improve patient outcomes.” (Pages 23-24, lines 311-316)

**Q3:** In the “Introduction” several statements are again pure speculative and should be omitted.

**A3:** We would like to thank the reviewer for the suggestion. The Introduction part has been revised to clearly state the unknown and why it is important to know. **Now it read** “Strokes in young adult patients are considered not uncommon, accounting for about 15% of all stroke patients.[1] It significantly affects the quality of life and has high economic impacts since affected individuals might become disabled before their most productive years, in contrast to strokes in older persons.[2] This background makes preventing stroke in young adults an urgent global public health concern. Because the etiologic spectrum of ischemic stroke in young adults is broader and more heterogeneous than in older people,[3] several investigations and tests are usually performed to evaluate all potential etiologies to select appropriate secondary stroke prevention, including standard, advanced, and specialized evaluation.[4]

Previous studies have been conducted to elucidate the risk factors, stroke outcomes and its predictors, and causes of stroke in young adult patients with the general agreement that uncommon causes and embolic sources of stroke might be the potential causes in individuals with younger age.[5-7] Importantly, there is no consensus on the definition of stroke in the young, and these studies ranged in age from 40 to 60 while determining young adults. Additionally, studies focused on different causes between gender and compared among young and younger adults are limited.

In the present study, we aimed to study the diagnostic tests of acute ischemic stroke (AIS) and transient ischemic attack (TIA) in young adults. We also investigated the causes and functional outcomes of stroke in this population using a large cohort of consecutive young adults with AIS or TIA. We hypothesized that our findings probably guided effective diagnostic methods to reduce the number of unnecessary investigations and establish appropriate stroke prevention.” (Page 4, lines 46-64)

**Q4:** Figure 4 should be omitted!

**A4:** We would like to thank the reviewer for the suggestion. Figure 4 is currently moved to a supporting information. **Now it read** “**S1 Fig. Diagnostic tests of ischemic stroke in young adults.** ANA, antinuclear antibody; CDUS, carotid doppler ultrasonography; CTA, computed tomography angiography; ESR, erythrocyte sedimentation rate; HIV, human immunodeficiency virus; MRA, magnetic resonance angiography; TEE, transesophageal echocardiography; TTE, transthoracic echocardiography.” (Page 30, lines 401-404) and S1_Fig.
