# Supplementary material for: The diagnostic tests and functional outcomes of acute ischemic stroke or transient ischemic attack in young adults: A 4-year hospital-based observational study
Source: PLoS One. 2023 Oct 4;18(10):e0292274. doi: 10.1371/journal.pone.0292274 (PMC10550126; doi:10.1371/journal.pone.0292274)
Supplement: Attachment — Submitted filename: Response to Reviewers.docx [file pone.0292274.s003.docx]

**Manuscript number: PONE-D-23-05766R1**

**Title: The diagnostic tests and functional outcomes of acute ischemic stroke or transient ischemic attack in young adults: A 4-year hospital-based observational study**

**Responses to Reviewers**

We would like to thank the academic editor and the reviewers for all the valuable comments and suggestions. We have revised the manuscript based on these constructive suggestions. We believe the revised manuscript is now greatly improved after the revisions have been made, as suggested by the reviewers and the editor.

**Responses to Academic Editor**

**Q1:** The authors are thanked for this submission to PLOS ONE. After a critical external peer review by three experts, I reinforced improving your paper's clarity and presentation and acknowledging study limitations according to the reviewer's concerns. Please see the attached reviewer comments detail below.

**A1:** We would like to thank the academic editor for the suggestion. The manuscript has been revised according to the reviewers’ comments.

**Q2:** Please review your reference list to ensure that it is complete and correct. If you have cited papers that have been retracted, please include the rationale for doing so in the manuscript text, or remove these references and replace them with relevant current references. Any changes to the reference list should be mentioned in the rebuttal letter that accompanies your revised manuscript. If you need to cite a retracted article, indicate the article’s retracted status in the References list and also include a citation and full reference for the retraction notice.

**A2:** We would like to thank the academic editor for the suggestion. The reference list has been thoroughly checked for its completeness and accuracy. No article cited in the manuscript has been retracted.

**Responses to Reviewer #1:**

**Q1:** The revised manuscript by Sakseranee et al improved and the authors tried to answer the questions and changed the manuscript. Although some concerns remain. The quality of data is limited. Therefore, a section “Limitations of the study” should be added. Here, all limitiations of the study should be mentioned.

**A1:** We would like to thank the reviewer for the helpful suggestions. We have revised the manuscript by adding a section on the limitations of the study, as suggested by the reviewer. **Now it read** “We acknowledge some limitations to our study. First, only 36% of our patients received a full complement of diagnostic testing, and the absence of disease for those patients with incomplete workups could not be guaranteed. In order to incorporate data to conduct a large cohort, we analyzed data from the entire cohort even though there are some missing data due to a retrospective study. Second, there was selection bias for some specialized evaluation, namely genetic testing, because this diagnostic workup was customarily conducted in patients with clinical clues or following other extensive studies. Lastly, because some diagnostic workups were performed in a small number in our cohort, such as TEE, representing a thin database of those investigations, we cannot state a conclusion on the yield of diagnostic tests or the value of TEE, especially in comparison with TTE in young adults.” (Pages 23-24, lines 311-320).

**Q2:** Again, only the patients with complete data set should be analyzed, or minimum all patients with TTE.

**A2:** We would like to thank the reviewer for the comment. In order to incorporate data to conduct a large cohort, we analyzed data from the entire cohort even though there are some missing data due to a retrospective study. We added this statement to the limitations of the study, as suggested by the reviewer.

**Q3:** How often was atrial fibrillation – a common cause for stroke - found in the cohort?

**A3:** We would like to thank the reviewer for the comment. We added the important issue regarding atrial fibrillation to the Discussion of the revised manuscript. **Now it read** “In contrast to the elderly, atrial fibrillation (AF), a common cause of ischemic stroke in older people, is less frequently observed in our cohort (5.7%).” (Pages 22, lines 271-271).

**Q4:** Table 1 and Table 5 do not clearly show sex-distribution within the subgroups.

**A4:** We would like to thank the reviewer for the comment. We acknowledge that gender disparity might affect the difference in stroke etiology between males and females. We, therefore, divided our cohort into two sub-cohorts, including gender and age cohorts (Fig 1). We revised the header row of Tables 1 and 5 for a more specific illustration, as suggested by the reviewer. (Table 1, Page 8-10 and Table 5, Page 17-19)

**Q5:** In general, it should be more emphasized, that the etiological clarification of the cause of a stroke especially in young patients is very important. Noninvasive tests like TTE and holter should be performed in ALL patients to get an explanation for a stroke.

**A5:** We would like to thank the reviewer for the valuable suggestion. We underscore the significance of the etiological elucidation of the cause of stroke, as suggested by the reviewer. **Now it read** “It is imperative to underline the significance of etiological explication in determining the cause of a stroke, particularly among young individuals. It is recommended that noninvasive diagnostic tests, such as Holter monitoring and TTE, be conducted generally in young stroke patients as part of the diagnostic workup for stroke etiology.” (Pages 23, lines 302-306).

**Responses to Reviewer #3:**

**Q1:** The authors examined the diagnostic tests, etiologies, and functional outcomes of acute ischemic stroke (AIS) and transient ischemic attack (TIA) in young patients in a single-center study of 244 patients over a 3-year period. In patients aged 18-40 years, the main reason was found to be "other determined etiologies" with 39.8%. The article is well prepared methodologically and linguistically. It is recommended to accept.

**A1:** We would like to thank the reviewer for the valuable comment. We sincerely appreciate your encouragement.

**Responses to Reviewer #4:**

**Q1:** I do appreciate the rewriting process conducted according to previous reviews as stroke in young is not uncommon problem and thus further publications are warranted. TEE, loop recorders and CTA utilization rate should be more encouraged in future publications.

**A1:** We would like to thank the reviewer for the valuable comment. We sincerely appreciate your encouragement.
